# A Retrospective Study on the Long-Term Outcomes of Stereotactic Ablative Radiotherapy Versus Sublobar Resection in Lung Cancer: Analysis From the Scottish Cancer Registry

**DOI:** 10.7759/cureus.69927

**Published:** 2024-09-22

**Authors:** Ghaith Qsous, Matthew McSorley, Thabbta Vianna, Christopher Fowler, George Korelidis, Zain Kabeer, Rory Moran, Anthony Chambers, Malcolm B Will, Vipin Zamvar

**Affiliations:** 1 Cardiothoracic Surgery, Royal Infirmary of Edinburgh, Edinburgh, GBR; 2 Thoracic Surgery, Royal Infirmary of Edinburgh, Edinburgh, GBR

**Keywords:** lung cancer, lung sabr, segmentectomy, stereotactic ablative radiotherapy, sublobar resection, survival outcomes, wedge resection

## Abstract

Introduction: A comparison between sublobar resection and stereotactic ablative radiotherapy (SABR) in the treatment of lung cancer is a trending topic. However, there is still a lack of strong randomized controlled trials on this subject. We believe that the National Cancer Registry can provide good insight into decision-making when comparing these two modalities of treatment.

Methods: The Scottish Cancer Network (SCAN) data were assessed retrospectively. These data included 2000 patients who had lung cancer between April 2013 and December 2022. We included 67 patients who had SABR (group 1) for early-stage lung cancer (T1-T2 N0) between January 2017 and December 2019 and 114 patients who had sublobar resection (group 2) for lung cancer (all comers) between April 2013 and December 2019.

Results: The average age in the SABR group was 74 years vs. 69 years in the sublobar group (P = 0.002). The overall recurrence was similar in the SABR group (29 patients) and the sublobar resection group (19 patients) (28.3% vs. 25.4%, respectively, P = 0.667). The overall survival (primary endpoint) was significantly better with patients who were treated with surgery than SABR (85.15 vs. 60.14 months, P = 0.006). The sublobar resection group showed an upgrade in staging after surgery compared to the preoperative stage on PET scan in the T stage (31 patients, 27.2%) and in the N stage (five patients, 4.3%). Patients who had SABR needed more CT scans for follow-up compared to the sublobar resection group (seven vs. four scans, respectively).

Conclusion: Sublobar lung resection should remain the preferable treatment for lung cancer in patients who are suitable for resection. Large randomized controlled trials are still needed to guide treatment in patients who are suitable for both options of treatment.

## Introduction

Lung cancer is the third most common cancer in the United Kingdom (UK), accounting for 13% of all new cancers. In Scotland, lung cancer is the second most common cancer, accounting for approximately 16% of all cancers diagnosed in men and women and it is the most common cause of cancer death in Scotland. Over the past few years, there have been efforts to improve early diagnosis of lung cancer such as lung cancer screening programs. As a result of an improvement in diagnostic methods, we will see an increase in the number of patients with early-stage lung cancer diagnoses. As longevity improves, we will have more elderly patients diagnosed with early-stage lung cancer [1-4].

In the last few years, there have been attempts to conduct large randomized controlled trials (RCTs) regarding the management of early-stage lung cancer comparing stereotactic ablative radiotherapy (SABR) with surgical treatment. The two main trials (ROSEL and STARS) could not recruit more than 54 patients and both trials were closed prematurely due to difficult recruitment and randomization. However, the results from a pooled analysis of these trials showed that SABR has better overall survival at one and three years compared to surgical treatment [5,6].

In the UK, there was an attempt to perform an RCT (SABRTooth) to compare SABR to surgery in peripheral early-stage lung cancer. In two years, they were able to randomize only 24 patients (10 in the surgical group and 14 for SABR) and the most common cause for nonparticipation was treatment preference, where approximately 40% of patients preferred SABR. They concluded that performing such a trial in the UK was unachievable [7,8].

Accordingly, with the lack of strong RCTs on this topic, we believe that the national cancer registry can provide some insight into decision-making when comparing these two modalities of treatment.

This article was previously presented as a meeting abstract at the World Society of Cardiovascular and Thoracic Surgery (WSCTS) Scientific Meeting on September 06, 2024.

## Materials and methods

The data were collected by the Scottish Cancer Network (SCAN) from the South East of Scotland over the last 10 years. South East Scotland has a population of 1.4 million people, which is 26% of Scotland's total population [9,10]. The SCAN data are not publicly accessible and we applied for permission from the SCAN audit office to get the data, review them, and publish the results.

The SCAN data were assessed retrospectively. The data included 2000 patients who had lung cancer between April 2013 and December 2022. We included 67 patients who had SABR (group 1) for early-stage lung cancer (T1-T2 N0) between January 2017 and December 2019 and 114 patients who had sublobar resection (group 2) for lung cancer (all comers) between April 2013 and December 2019. It is worth mentioning that SABR was started in this region in 2017 and until December 2022, there were 353 patients in this period treated with SABR.

In the SABR group, between 2017 and December 2022, there were 150 patients treated with SABR. We excluded those with a performance status (PS) of more than 1 but in the surgical group, we included all comers (PS 0, 1, and 2). By doing this in the inclusion criteria, we aimed to select more fit patients in the SABR group who could be candidates for surgery to give this group of patients some privilege over the surgical patients. In exclusion criteria, we also excluded any patients with N2, T3 disease, stage four of lung or other cancer, and any patient who died within one year as the main outcome of this study is to identify the long-term outcomes (three- and five-year survival and recurrence). After the exclusion of these patients, we had 67 patients in the SABR group.

Only patients with stage 1 non-small cell lung cancer were treated by SABR. The treatment protocol for SABR was 54 Gy in three fractions, 55 GY in five fractions, or 60 Gy in eight fractions.

The information missed from the SCAN data was extracted from the digital records of the patients.

In the statistical analysis, the independent t-test was employed to investigate the mean difference in patients' age based on the study group. The chi-square test was used to explore the association between categorical data. The Kaplan-Meier test was utilized to investigate disease-free survival and overall survival between the two groups. The log-rank test was used to determine if the survival analysis was statistically significant between the two groups.

## Results

The average age in the SABR group was 74 ± 7.9 years vs. 70 ± 8.6 years in the sublobar group (P = 0.002). Patients who were treated with SABR had significantly better performance status (0 and 1) compared to those who were treated with surgery (all comers - PS 0, 1, and 2). Out of 114 patients who had surgery, 79 (69.3%) had segmentectomy and 35 (30.7%) had wedge resection. Most patients had minimally invasive surgery (video-assisted thoracoscopic surgery) (Table 1).

**Table 1:** Patients characteristics and surgical outcomes. The independent t-test was employed to investigate the mean difference in patients' age based on the study group. The chi-square test was used to explore the association between categorical data. VATS: video-assisted thoracoscopic surgery; NSCLC: non-small cell lung cancer.

Variable	Sublobar resection (114 patients)	Stereotactic ablative radiotherapy (SABR) (67 patients)	P-value
Age	69.9 ± 8.6	74.0 ± 7.9	0.002
Sex:	-	-	0.368
Male	40 (35%)	28 (41.8%)	-
Female	74 (65%)	39 (58.2%)	-
Performance status:	-	-	<0.001>
0	53 (46.5%)	9 (13.5%)	-
1	50 (43.9%)	58 (86.5%)	-
2	9 (7.8%)	0	-
3	2 (1.8%)	0	-
Pre-treatment histology:	-	-	<0.001>
Malignant	47 (41.3%)	16 (23.8%)	-
Inconclusive/negative	31 (27.2%)	5 (7.4%)	-
No biopsy	36 (31.5%)	46 (68.6%)	-
Pre-treatment PET scan T stage:	-	-	0.264
T1	101 (88.7%)	60 (89.5%)	-
T2	9 (7.8%)	7 (10.5%)	-
T3	4 (3.5%)	0	-
Pre-treatment PET scan N stage:	-	-	0.161
N0	108 (94.8%)	67 (100%)	-
N1	4 (3.5%)	0	-
N2	2 (1.7%)	0	-
Surgical approach:	-	-	Not computed
Open	7 (6.1%)	-	-
VATS	107 (93.9%)	-	-
Volume of resection:	-	-	Not computed
Segmentectomy	79 (69.3%)	-	-
Wedge resection	35 (30.7%)	-	-
Post-surgical histology:	-	-	Not computed
Squamous cell carcinoma (SCC)	25 (21.9%)	-	-
Adenocarcinoma	71 (62.4%)	-	-
Carcinoid tumor	8 (7%)	-	
Other NSCLC	10 (8.7%)	-	
Post-surgical T stage:	-	-	Not computed
T1	72 (63.1%)	-	-
T2	38 (33.3%)	-	-
T3	3 (2.5%)	-	-
T4	1 (1.1%)	-	-
Post-surgical N stage:	-	-	Not computed
N0	89 (78%)	-	-
N1	6 (5.3%)	-	-
N2	4 (3.5%)	-	-
Nx	15 (13.2%)	-	-
Surgical margins:	-	-	Not computed
R0	103 (90.4%)	-	-
R1	11 (9.6%)	-	-
Upstage after surgery:	-	-	Not computed
T upstage	31 (27.2%)	-	-
N upstage	5 (4.3%)	-	-

There were 31 (68.8%) patients who had SABR and were diagnosed with moderate to severe chronic obstructive pulmonary disease (COPD). Regarding pulmonary function test (PFT) in patients who had SABR, 54.7% of them presented with forced expiratory volume in one second (FEV1) > 60%, 35.7% presented with FEV1 = 60-40%, and 9.5% presented with FEV1 less than 40%.

When assessing fitness for surgery, only nine (13.5%) were assessed by surgeons in the clinic, and the remaining 58 (86.5%) patients were not assessed.

The most common documented reason for wedge resection in the surgical group was the poor PFT in the surgical candidates (Table 2).

**Table 2:** Reasons for performing a wedge resection.

Reasons for wedge resection	Number of patients (n = 35) (%)
Poor pulmonary function test	10 (28.5%)
Diagnostic	6 (17.1%)
Technical difficulties	7 (20%)
Previous bilobectomy	1 (2.8%)
Not documented	11 (31.4%)

In the SABR group, most of the patients were referred from the multi-disciplinary team (MDT) meetings to have SABR directly, and only 11 (16.4%) of the patients were referred for surgery but they chose to have SABR (Table 3).

**Table 3:** Reasons for choosing SABR as a radical treatment. SABR: stereotactic ablative radiotherapy; MDT: multi-disciplinary team.

Reason for SABR	Number of patients (n = 67) (%)
Patient's choice	11 (16.4%)
Respiratory physician's decision	4 (6%)
MDT's decision	52 (77.6%)

The surgical group showed an upstaging on T (27.2%) and N stage (4.3%) after surgery, compared to the preoperative clinical stage. In patients who underwent surgery, 10 (9.6%) patients were reported with R1 surgical margin, and of these, seven patients underwent a wedge resection and three had an anatomical segmentectomy and they were not referred for radical treatment (Table 1).

The overall survival (primary endpoint) was significantly better with patients who were treated with surgery compared to SABR (85.15 vs. 60.14 months, P = 0.006). The one-year survival was similar between groups (approximately 97%), but the three and five-year survival outcomes were better in patients treated with surgery (83.3% and 62.3% vs. 77.6% and 49.2%, respectively) but without statistical significance (Table 4).

**Table 4:** Oncological outcomes. The number of patients who had a recurrence in the sublobar group was 29 out of 114 patients. Seven out of 29 patients had recurrence in the first year and 15 out of 29 patients had recurrence within three years. The number of patients who had a recurrence in the SABR group was 19 out of 67 patients. Four out of 19 patients had recurrence in the first year and 10 out of 29 patients had recurrence within three years. The Kaplan-Meier test was utilized to investigate disease-free survival and overall survival between the two groups. The log-rank test was used to determine if the survival analysis was statistically significant between the two groups.

Variables	Sublobar resection (114 patients)	Stereotactic ablative radiotherapy (SABR) (67 patients)	P-value
Recurrence	29 (25.4%)	19 (28.3%)	0.667
Disease-free survival	28.2 months	26 months	0.443
Overall survival	85.15	60.14	0.006
1-year survival	110 (96.4%)	65 (97%)	0.849
3-year survival	95 (83.3%)	52 (77.6%)	0.341
5-year survival	63 (62.3%)	32 (49.2%)	0.329
Recurrence within 1 year	7 (24.1%), 7 out of 29	4 (21%), 4 out of 19	0.803
Recurrence within 3 years	15 (51.7%), 15 out of 29	10 (52.6%), 10 out of 19	0.951
Number of CT scans in follow up	4	7	0.845

**Figure 1:**
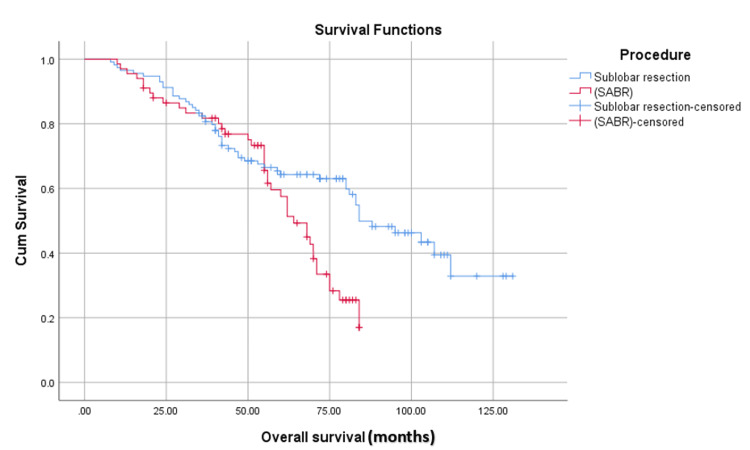
Comparing the overall survival in months using the Kaplan-Meier test. SABR: stereotactic ablative radiotherapy.

In Figure 1, we can see the trend of survival time between the two groups. Despite both groups showing a decline in survival probability over time, the resection group maintained a higher cumulative survival probability compared to the SABR group.

The overall recurrence was higher in patients who were treated with SABR than surgery but without statistical significance (28.3% vs. 25.4%, P = 0.667). However, the recurrence within the first year was slightly higher in patients who had surgery than SABR (24.1% vs. 21%, P = 803), which could be explained by the higher rate of R1 resection (Table 4).

**Figure 2:**
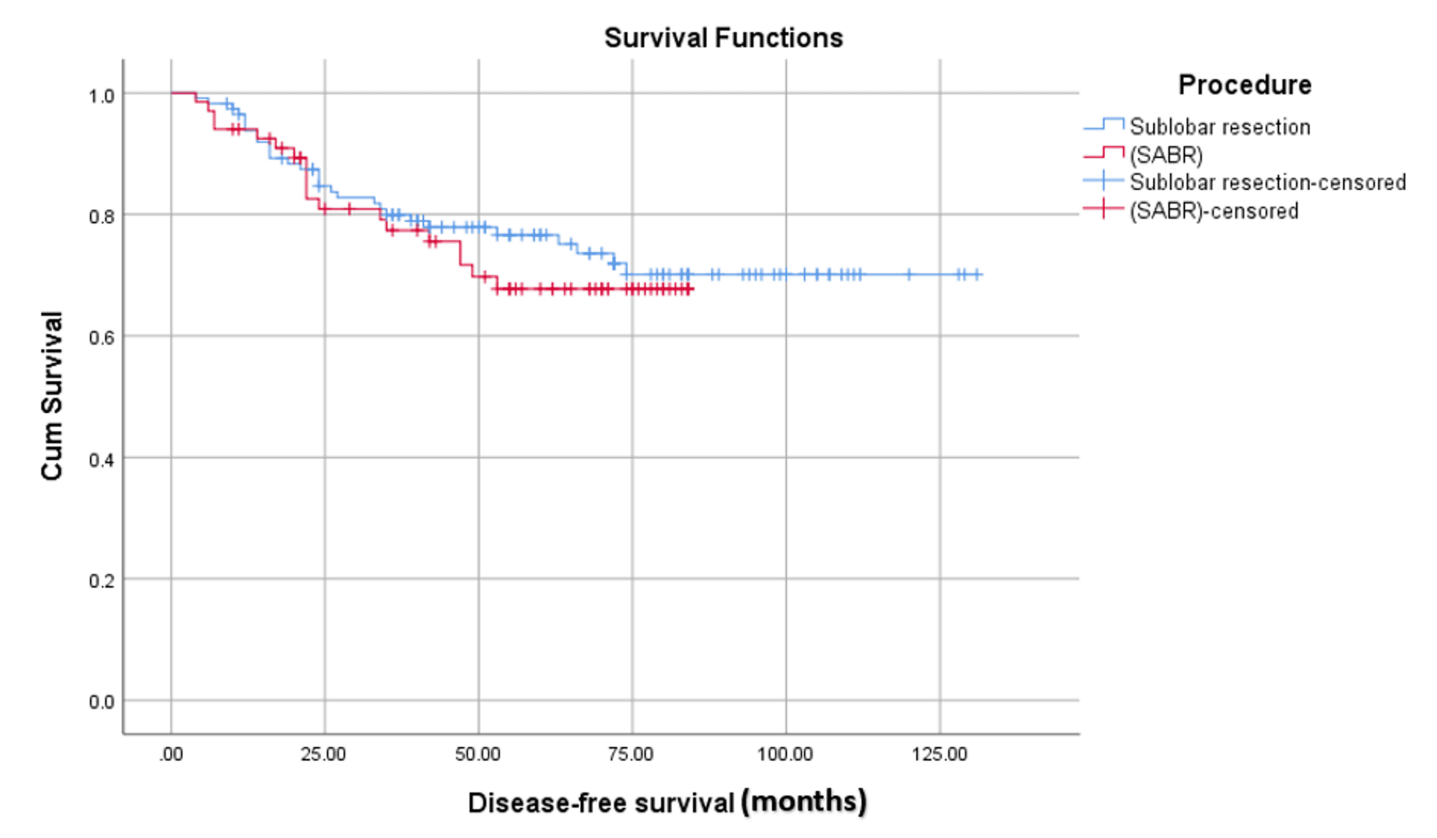
Comparing disease-free survival in months using the Kaplan-Meier test. SABR: stereotactic ablative radiotherapy.

The disease-free survival was similar in both groups (28.2 vs. 26 months, P = 0.443) (Figure 2).

Regarding surveillance, CT scan was used more frequently (approximately two-fold more) in patients who had SABR than in surgery who were followed up with chest X-ray. It is worth mentioning that we were using chest X-rays as a surveillance method after lung cancer resection but now we moved forward to CT scan as part of our surgical surveillance (Table 4).

## Discussion

For many decades, lobectomy was the standard of care for lung cancer, and the first RCT comparing lobectomy and sublobar resection (segmentectomy and wedge) conducted in 1995 by Ginsberg et al. showed that the recurrence was significantly higher in the sublobar group and there was a higher rate of cancer death [11]. Usually, the debate was regarding the best treatment for patients who are borderline fit and have poor PFT. The argument was that wedge resection is not adequate as oncological resection and SABR should be the preferred option. However, in the last few years, there were two large RCTs (JCOG 0802 and CALGB) where they compared lobectomy to sublobar resection, and the results of both trials showed that sublobar resection is not inferior to lobectomy in selected patients, and the results had a significant effect on the practice of thoracic surgery. The JCOG 0802 trial showed that segmentectomy was not inferior to lobectomy in clinical stage IA non-small cell lung cancer (NSCLC) (tumor < or equal 2 cm, consolidation-to-tumor ratio > 0.5), and in the CALGB trial, in the sublobar group, there were 60% of cases who underwent wedge resection and they proved that wedge resection and segmentectomy are not inferior to lobectomy in same selected patients [12-14]. These results gave thoracic surgeons strong evidence to use sublobar resection in patients with borderline fitness and poor PFT.

Stares et al., in 2023, published a study based on the same cancer registry that we used in our study (SCAN data) and they compared SABR to other treatment modalities of early-stage lung cancer, but in their study, they included in the SABR group all performance status (0-2). They found that surgery was associated with significantly better outcomes (overall survival) in comparison to SABR and conventional radical radiotherapy. However, SABR was significantly better compared to conventional radical radiotherapy and they concluded that the use of SABR in this region of Scotland appears to have enhanced the selection of surgical patients and increased the proportion of patients receiving a radical therapy [15].

Paul et al., in 2016, published a national population-based study from the United States to compare SABR and sublobar resection in early-stage lung cancer. They found that patients who underwent surgery for a tumor size of 5 cm or less had improved cancer-specific survival over SABR (P < 0.001). The estimated cancer-specific survival at three years was 80.0% and 90.3%, respectively. However, there was no significant difference between groups if the tumor size was 2 cm or less. They concluded that surgery is better, especially in large tumors [16].

It is always a challenge when surgeons assess patients with borderline fitness and PFT who can be candidates for radical surgical treatment.

Most of our patients who had SABR did not have proper assessment by surgeons, and they did not have a pulmonary rehabilitation program that may improve their fitness. Bibo et al., in 2021, reviewed the best evidence on the role of preoperative pulmonary rehabilitation in patients who are a candidate for lung resection and they found that these patients had fewer postoperative pulmonary complications, shorter lengths of stay, and improved six-minute walking tests [17]. Even the benefit of home-based preoperative pulmonary rehabilitation was studied by Saito et al., where patients who planned to have lung resection underwent pulmonary rehabilitation exercises for two to four weeks before surgery and the results showed that these patients had significantly fewer postoperative complications (Clavien-Dindo class I) [18]. Accordingly, we encourage hospitals to organize preoperative pulmonary rehabilitation programs for borderline patients with poor PFT who can be candidates for curative lung resection.

The Healthcare Quality Improvement Partnership (HQIP) in the UK published the report of Lung Cancer Clinical Outcomes Publication (LCCOP) in 2020. They mentioned the best practices in certain aspects, and one of them was for the Oxford unit because they achieved high resection and survival rates of lung cancer. One of the causes of achieving this success was that they had two thoracic surgeons attending the MDT in person, providing an internal second surgical opinion. This approach maximizes surgical opportunities for borderline operable patients [19]. We encourage this practice to be adopted in all units and we believe that surgeons should meet borderline operable patients in person to have better assessment and evaluation, which may increase the rate of patients having curative surgical resection.

This topic is challenging, especially with the lack of strong evidence. We look forward to seeing the results of the ongoing RCT, i.e., the STABLE-MATES trial, which compares SABR with sublobar resection in medically high-risk patients with stage I NSCLC. The inclusion criteria are peripheral proven malignant tumor with a size of 4 cm or less and nodal negative. The primary endpoint is the overall survival and the secondary endpoints are the progression-free survival and toxicity [20].

In this study, despite the strict selection criteria in the SABR group where we aimed to select the most fit patients and the majority had acceptable PFT, the results show that sublobar resection still gives better long-term outcomes in survival. Also, the upgrading in the T and N stages is very important as these patients may be candidates for adjuvant treatment. With the evolution in immunotherapy and targeted therapy in lung cancer, these patients will have more choices in adjuvant treatment. Accordingly, borderline patients must be reviewed by surgeons in person and offer preoperative rehabilitation and should mention to them the possibility of upstaging after surgery, which may make them candidates for further treatment after surgery.

The limitation of our study is that it is a retrospective study with a relatively small number of patients in the SABR group and no available data about the cause of death.

## Conclusions

Surgical treatment should remain the standard treatment for lung cancer in patients who are suitable for resection. Borderline patients must have multi-disciplinary assessment before making a decision that they are not fit for surgery. Large RCTs are needed to guide treatment in patients who are suitable for both options of treatment.
